# Validation of the spiritual distress assessment tool in older hospitalized patients

**DOI:** 10.1186/1471-2318-12-13

**Published:** 2012-03-29

**Authors:** Stefanie Monod, Estelle Martin, Brenda Spencer, Etienne Rochat, Christophe Büla

**Affiliations:** 1 Service of Geriatric Medicine & Geriatric Rehabilitation, University of Lausanne Medical Center (CHUV), 1011 Lausanne, Switzerland; 2 Institute of Social and Preventive Medicine (IUMSP), University Hospital Center and University of Lausanne, Bugnon 17, 1005 Lausanne, Switzerland; 3 Chaplaincy Service, University of Lausanne Medical Center (CHUV), 1011 Lausanne, Switzerland

## Abstract

**Background:**

The Spiritual Distress Assessment Tool (SDAT) is a 5-item instrument developed to assess unmet spiritual needs in hospitalized elderly patients and to determine the presence of spiritual distress. The objective of this study was to investigate the SDAT psychometric properties.

**Methods:**

This cross-sectional study was performed in a Geriatric Rehabilitation Unit. Patients (N = 203), aged 65 years and over with Mini Mental State Exam score ≥ 20, were consecutively enrolled over a 6-month period. Data on health, functional, cognitive, affective and spiritual status were collected upon admission. Interviews using the SDAT (score from 0 to 15, higher scores indicating higher distress) were conducted by a trained chaplain. Factor analysis, measures of internal consistency (inter-item and item-to-total correlations, Cronbach α), and reliability (intra-rater and inter-rater) were performed. Criterion-related validity was assessed using the Functional Assessment of Chronic Illness Therapy-Spiritual well-being (FACIT-Sp) and the question "Are you at peace?" as criterion-standard. Concurrent and predictive validity were assessed using the Geriatric Depression Scale (GDS), occurrence of a family meeting, hospital length of stay (LOS) and destination at discharge.

**Results:**

SDAT scores ranged from 1 to 11 (mean 5.6 ± 2.4). Overall, 65.0% (132/203) of the patients reported some spiritual distress on SDAT total score and 22.2% (45/203) reported at least one severe unmet spiritual need. A two-factor solution explained 60% of the variance. Inter-item correlations ranged from 0.11 to 0.41 (eight out of ten with P < 0.05). Item-to-total correlations ranged from 0.57 to 0.66 (all P < 0.001). Cronbach α was acceptable (0.60). Intra-rater and inter-rater reliabilities were high (Intraclass Correlation Coefficients ranging from 0.87 to 0.96). SDAT correlated significantly with the FACIT-Sp, "Are you at peace?", GDS (Rho -0.45, -0.33, and 0.43, respectively, all P < .001), and LOS (Rho 0.15, P = .03). Compared with patients showing no severely unmet spiritual need, patients with at least one severe unmet spiritual need had higher odds of occurrence of a family meeting (adjOR 4.7, 95%CI 1.4-16.3, P = .02) and were more often discharged to a nursing home (13.3% vs 3.8%; P = .027).

**Conclusions:**

SDAT has acceptable psychometrics properties and appears to be a valid and reliable instrument to assess spiritual distress in elderly hospitalized patients.

## Background

Spirituality is an important component of quality of life, and a resource in patients coping with illness [1,2]. In elderly persons, spirituality is probably a significant factor when facing disability and approaching death [3-9]. While spirituality was associated with better mental and physical health in several studies [10,11], other studies have also suggested that some negative aspects of spirituality (e.g., "low spiritual well-being" or "religious struggle"), might be associated with worse health outcomes [11-14].

All these observations support the growing consensus about the need to better integrate the spiritual dimension into hospital care [15-17]. However, promoting such integration requires an appropriate assessment of patient spirituality, the definition of conditions for spiritual interventions, and good evidences that specific interventions to address spiritual issues would improve patient care [16-18]. Models that address these gaps are still lacking.

Numerous instruments have been developed to assess patients' spirituality [19], most focusing on measurement of attitudes and behaviours. Nevertheless, these instruments provide little information on the patient's current intimate feelings related to spirituality (e.g. feeling peacefulness or meaning in life), limiting their use to determine a patient's *spiritual state*. However, measuring the spiritual state, and particularly the lower end of the patient's spiritual state, namely *spiritual distress*, is probably the most appropriate way to assess patient spirituality within the hospital setting. This measure would serve to determine the need for specific interventions. In a recent systematic review [19], only two out of 35 instruments appeared adequate to assess a patient's current spiritual state [20,21]. Moreover, these two instruments were developed to measure spiritual well-being rather than spiritual distress: "low spiritual well-being" is not necessarily equivalent to spiritual distress.

**Figure 1:**
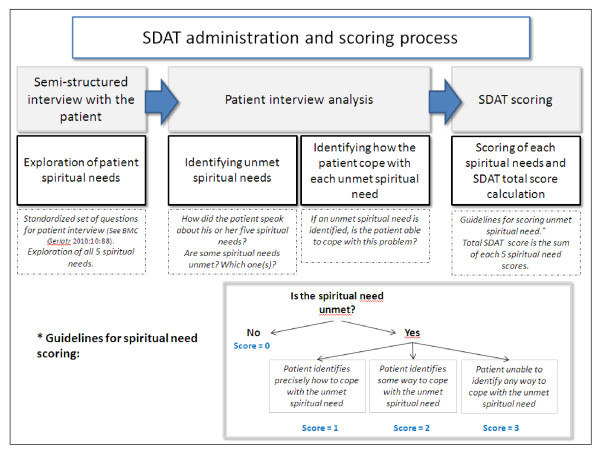
**SDAT administration and scoring process**.

The Spiritual Distress Assessment Tool (SDAT) was developed to address the need for a valid instrument specifically designed to assess spiritual distress in hospitalized elderly patients. The hypothesis was made that *spiritual distress *arises from *unmet *spiritual needs. The greater the degree to which a spiritual need remains unmet, the greater the disturbance in spiritual state and the greater the level of spiritual distress experienced by the patient. Within this conceptual framework, the SDAT was developed in three stages. First, a conceptual model of spirituality, the Spiritual Needs Model, was defined [22]. In this model, spirituality in hospitalized elderly persons is defined as a multidimensional concept that includes four dimensions: Meaning, Transcendence, Values and Psycho-social Identity. Related spiritual needs were systematically defined for each dimension. The dimensions and their related needs are presented in Table 1. Second, the SDAT instrument was developed on the basis of this model [23]. A standardised set of questions to be used in a semi-structured interview performed by a chaplain has been specifically defined. Moreover, a structured assessment procedure to identify unmet spiritual needs and score the degree to which spiritual needs remain unmet was successively developed. The overall process for SDAT administration and scoring is presented in Figure 1 and an example of SDAT scoring is provided in Table 2. Finally, face validity and acceptability of the SDAT instrument were evaluated in chaplains experienced in hospital pastoral care. Results confirmed very good face validity and showed high acceptability of the SDAT [23].

**Table 1:** The Spiritual Needs Model

Dimensions	Definition of dimension	Definition of need related to dimension
**Meaning**	The dimension that provides orientation to an individual's life and promotes his or her overall life balance.	**The Need for life balance** The need to rebuild a new life balance and the need to learn how better to cope with illness or disability.

**Transcendence**	The anchor point exterior to the person; the relationship with an external foundation that provides a sense of grounding.	**The Need for connection** The need for connection with his or her existential foundation and the need for Beauty (aesthetic sense).

**Values**	The system of values that determines goodness and trueness for the person; it is made apparent in the person's actions and life choices.	**Value 1: The Need for values acknowledgement** The need that health professionals know and respect one's values.
		
		**Value 2: The Need to maintain control** The need to understand and to feel included in decision-making processes and to be associated with health professionals' decisions and actions.

**Psycho-social Identity**	The patient's environment; those elements, such as society, caregivers, family, and close relationships that together make up the person's singular identity.	**The Need to maintain identity** The need to be loved, to be heard, to be recognized, to be in touch, to have a positive image of oneself and to feel forgiven.

**Table 2:** Example of need for life balance scoring

Patient interview	Interview analysis		SDAT scoring
**Exploration of patient *Need for life balance***	**Identifying unmet *Need for life balance***	**Identifying how the patient cope with the unmet *Need for life balance***	**Scoring of *Need for life balance***

*A 81 years old woman, hospitalized for rehabilitation after a hip fracture, says: "I know that I will recover and that everything will be fine... I'm sure that I will be able to do with"*.	No unmet spiritual need is identified	Not appropriate	**Score = 0** No evidence of unmet Need for life balance

*A 81 years old woman, hospitalized for rehabilitation after a hip fracture, says: "This fracture will change a lots of things in my life. But I know I have the resources to deal with it... I will recover and everything will be fine."*	An unmet spiritual need is identified: This patient says "*This fracture will change a lots of things in my life"*	This patient identifies how to cope with this unmet spiritual need. She says "*I know I have the resources to deal with it... I will recover and everything will be fine."*	**Score = 1** Some evidence of unmet Need for life balance

*A 81 years old woman, hospitalized for rehabilitation after a hip fracture, says: "This fracture will change a lots of things in my life. I know that I have some resources to deal with it... but I have more pain than before and I don't really know how it will be at home..."*	An unmet spiritual need is identified: This patient says "*This fracture will change a lots of things in my life"*	This patient identifies some way to cope with this unmet spiritual need. She says "*I know that I have some resources to deal with it..." *However, she still has some doubts. She says: *"I have more pain than before and I don't really know how it will be at home..."*	**Score = 2** Substantial evidence of unmet Need for life balance

*A 81 years old woman, hospitalized for rehabilitation after a hip fracture, says: "This fracture will change a lots of things in my life. I have more pain than before... I feel that I am very down... I can't imagine any future... I don't know what to do..."*	An unmet spiritual need is identified: This patient says "*This fracture will change a lots of things in my life"*	This patient is not able to identify any way to cope with the unmet spiritual need. She says " *I can't imagine any future... I don't know what to do..."*	**Score = 3** Evidence of severe unmet Need for life balance

The aim of the current study was to investigate the psychometric properties of the SDAT in elderly hospitalized patients. Specifically, the objectives were to investigate the structure of the SDAT and to determine its internal consistency, intra-rater reliability, and inter-rater reliability. Criterion-related validity was also assessed using the Functional Assessment of Chronic Illness Therapy- Spiritual Well Being (FACIT-Sp) [20] and the question "Are you at peace?" [24]. In addition, based on the hypothesis that spiritual distress would be correlated with depressive symptoms and with difficulties in discharge planning, concurrent validity of the SDAT was assessed with the Geriatric Depression Scale (GDS) [25] and the occurrence of a family meeting to define discharge disposition, respectively. Finally, predictive validity was investigated using rehabilitation length of stay and nursing home discharge as outcome measures.

## Methods

### Setting and population

This study was performed in the post-acute Rehabilitation Unit of the Service of Geriatric Medicine, University of Lausanne Medical Center, Switzerland. In this setting, around 80% of patients report a Judaeo-Christian religious background.

Participants were patients aged 65 years and over consecutively admitted to the Unit over a 6-month period. Subjects were not eligible if suffering from significant cognitive impairment (defined as Mini Mental State Exam-MMSE [26] score < 20), unable to speak French or considered too ill to complete the interview (medically unstable or with uncontrolled symptoms such as pain, dyspnea, etc.).

The sample size was calculated to achieve a sufficient statistical power (80%) for investigation of the predictive validity. Spiritual distress prevalence was estimated based on results from a pilot study performed in the same geriatric unit where 61% of patients were found to have some spiritual distress [27]. Assuming a conservative prevalence of spiritual distress (50%) in this study, a sample size of 198 will be needed to achieve a 80% power (at α = .05) to detect a 1.0 day difference in length of stay between patients with and without spiritual distress.

**Figure 2:**
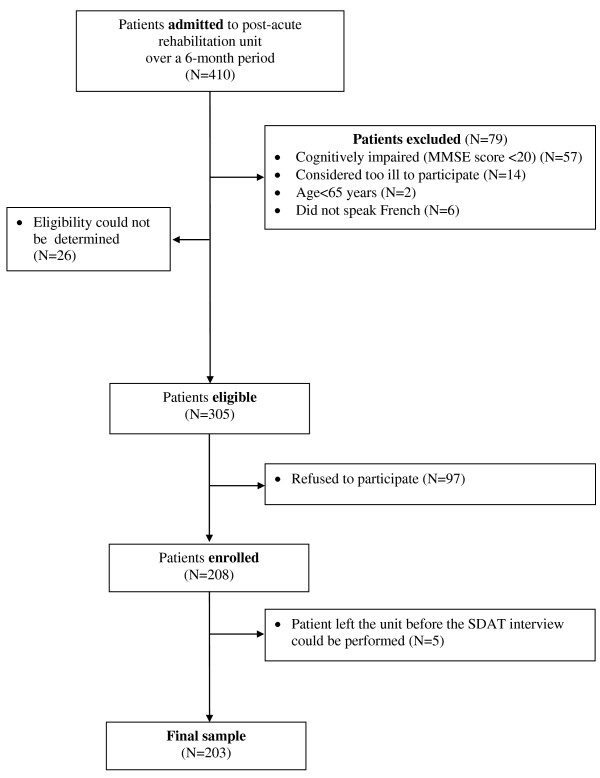
**Study flow-chart**.

The study flow-chart is represented in Figure 2. Over the 6-month inclusion period, 305 of the 410 patients admitted to the Unit were found eligible. Within 3 days of admission, patients were asked by a research assistant to participate in the study. Ninety seven of the 305 (31.8%) eligible patients refused and five additional patients left the Unit before the SDAT interview could be performed, leaving a final sample of 203 patients for analysis.

The study was approved by the institutional ethical committee and written informed consent was obtained from all study participants.

### Data collection

Data on demographics, living arrangements, observed performance in basic activities of daily living [28] (Basic ADLs), cognitive status (MMSE [26]) and affective status (15-item GDS [25]) were systematically collected upon admission. Occurrence of a family meeting for discharge planning, length of rehabilitation stay (LOS) and destination at discharge were obtained from the hospital administrative database.

All included participants were interviewed by the research assistant to complete two instruments:

#### The FACIT-Sp [20]

This 12-item scale includes two subscales that measure meaning and faith. Total scores range from 0 to 48, a higher score indicating higher spiritual well-being. Authorization to use the FACIT-Sp was obtained from the FACIT organization.

#### The single question "Are you at peace?"[24]

This question has been strongly correlated with emotional and spiritual well-being in patients with serious illnesses. Participants were asked to answer to this question on a visual analog scale, ranging from 0 to 10, a higher score indicating a higher level of peacefulness.

### SDAT interviews

Within three days after the initial assessment, a chaplain (ER) confirmed participants' consent to complete the SDAT interview. All participants agreed. The SDAT was administered by the chaplain according to the following procedure that has been previously described (Figure 1) [23]. First, a 20 - 30 minute semi-structured patient interview is conducted by the chaplain. During this interview, the chaplain invites the patient to speak about what she or he is currently experiencing during hospitalization. The chaplain uses the standardized set of questions only if the patient does not spontaneously speak about each one of the defined spiritual needs and, at the end of the interview, checks that all five different spiritual needs have been investigated. Second, after completion of the interview, the chaplain analyses how the patient has spoken about his or her five spiritual needs, and determines whether each spiritual need is met or not. He also identifies how the patient is coping with each unmet spiritual need. Third, according to this analysis and the guidelines for spiritual needs scoring (Figure 1 and Table 2), the chaplain determines the degree to which each spiritual need is unmet, and scores this on a 4-point Likert scale ranging from 0 (no evidence of unmet spiritual need) to 3 (evidence of severe unmet spiritual need). The global score of spiritual distress is calculated as the sum of each spiritual need score and may therefore range from 0 (no distress) to 15 (severe distress). Spiritual distress is defined as a score ≥ 5 because this cut-off corresponds to a range of situations with unmet needs considered as significant either in terms of severity (e.g., *one severe unmet spiritual need *in one dimension combined with some unmet spiritual need in two other dimensions) or in terms of extent (e.g., *unmet spiritual need in all five dimensions*).

### Psychometric assessment

**Figure 3:**
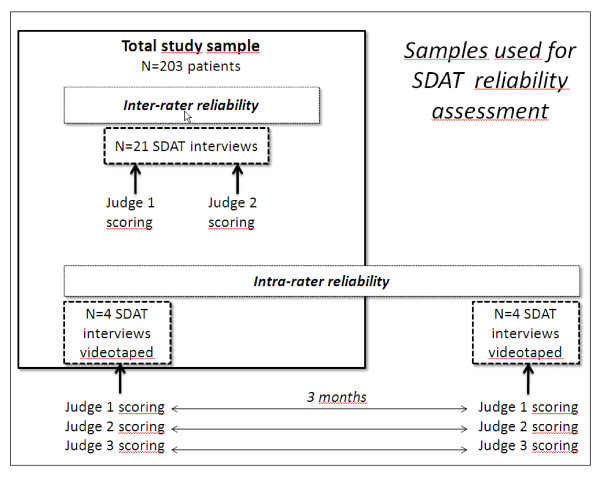
**Reliability assessment: overall procedure**.

The overall procedure for SDAT reliability (intra-rater and inter-rater) assessment is summarized in Figure 3.

#### Intra-rater reliability

Intra-rater reliability measures the consistency between SDAT scorings performed on two separate occasions but involving the same rater. This way to perform test-retest reliability was adopted because it was considered inappropriate to repeat a complete SDAT interview and perform a test-retest with the same patient. Videotaped SDAT interviews were performed to assess intra-rater reliability of SDAT scoring. Seven randomly chosen participants were requested permission to videotape their interview and four gave consent. In addition to the chaplain who conducted the SDAT interview (ER), one of the co-authors (SM) and another chaplain (also trained to use the SDAT) scored the four SDAT interviews twice, 3 months apart. All three judges were blinded to other judges scoring, and were not reminded of their own initial scoring when repeating their assessment. Variations in measurements of the same items of the SDAT, with the same judge and under the same conditions, were assessed after a 3-month interval.

#### Inter-rater reliability

Inter-rater reliability was assessed in a random sample (N = 21) of elderly patients included in the study. Written consent was also obtained from all these patients. For this analysis, the most experienced chaplain conducted the interview (ER) in the presence of one other chaplain trained to use the SDAT. Both performed a separate and blinded scoring of the SDAT.

### Statistical analysis

Characteristics of participants were described using simple descriptive statistics and compared to those of patients who refused to participate in the study.

Factor analysis of the SDAT, verified by Kaiser-Meyer Olkin measure and adequacy of uniqueness, was performed using principal component analysis with varimax rotation.

Internal consistency was assessed using Pearson's coefficient from inter-item and item-to-total correlation analyses. Internal reliability was assessed using Cronbach α coefficient.

Intra-rater reliability was assessed by intraclass correlation coefficients (one-way analysis of variance with random effects) between SDAT scores at test and re-test 3 months later (three judges, N = 4 SDAT interviews). Correlations were calculated, for each judge, between test and retest SDAT scoring.

Intraclass correlations coefficients were also used to determine inter-rater reliability of SDAT scores (two judges, N = 21 SDAT interviews). Cohen's kappa was used to calculate agreement between the two raters about the presence or absence of spiritual distress.

Criterion-related validity was assessed using Spearman's Rho from correlation analyses with the FACIT-Sp score and the single question "Are you at peace?" both used as continuous variables.

Concurrent validity was assessed using Spearman's Rho from correlation analyses with the GDS score used as a continuous variable. Bivariate logistic regression, then multivariate logistic regression adjusted for age, gender, functional and depressive status, were performed to obtain odds of occurrence of a family meeting for discharge planning.

Finally, to determine predictive validity, bivariate analyses as well as robust multivariate regression controlling for age and gender were performed to identify the association between SDAT and rehabilitation outcomes (length of stay, nursing home discharge).

Statistical analyses were performed using Stata (Version 11.0).

## Results

### Population characteristics

Characteristics of participants and comparison with patients who refused to participate are summarized in Table 3. Mean age of participants was 81.4 ± 7.1 years. Overall, 69.5% were women and 56.3% were living alone. Mean Basic ADL at admission was 3.4 ± 1.5. Prevalence of cognitive impairment and depressive symptoms was 15.8% and 14.7% respectively. No significant differences in these measures were found between participants and patients who refused to participate in the study.

**Table 3:** Characteristics of participants and comparison with patients who refused to participate

Characteristics	Study sample (N = 203)	Patients who refused to participate (N = 97)	P-val Wilcoxon or Chi2
Mean Age (years)	81.4 ± 7.1	80.8 ± 6.9	0.361

Women (%)	69.5	66.0	0.545

Living alone (%)	56.3	58.5	0.719

Cognitive impairment* (%)	15.8	16.5	0.890

Depressive Symptoms^§ ^(%)	14.7	12.4	0.594

Basic ADL at admission^¥^	3.4 ± 1.5	3.1 ± 1.5	0.195

* Cognitive impairment defined as a MMSE score < 24^26^

^§ ^Depressive symptoms defined as GDS score ≥ 6 ^25^

^¥ ^Basic ADL from Katz ^28^

**Figure 4:**
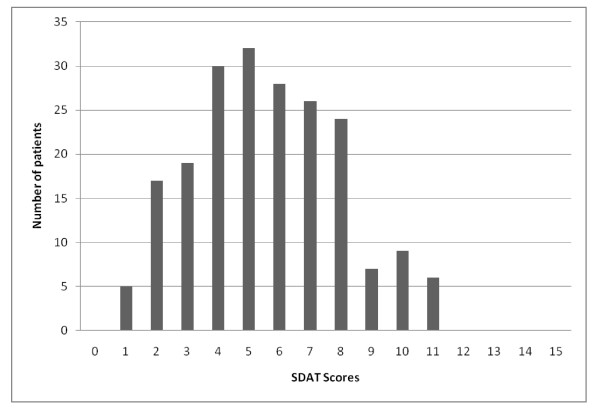
**Distribution of Spiritual Distress Assessment Tool (SDAT) scores in the study population**. Scores may range from 0 (no spiritual distress) to 15 (severe spiritual distress).

Distribution of SDAT total scores are presented in Figure 4. Overall, 65.0% (132/203) of the patients reported some spiritual distress on the SDAT total score, 22.2% (45/203) reported one severe unmet spiritual need in at least one dimension, and 28.6% (58/203) reported some distress on all five spiritual needs. All patients reported some distress on the Need for Life balance. Moreover, the Need for Life balance accounted for more than half of the severely unmet spiritual needs (59.6%).

### Factor analysis

Results of factorial analysis are presented in Table 4. A two factor solution explained 60% of the variance and clearly distinguished between the Meaning, Transcendence and Identity related needs (first factor), and needs related to the Values dimension (second factor).

**Table 4:** Factor analysis

Dimension	Factor1	Factor2	*Uniqueness*
Meaning	**0.611**	0.233	*0.572*

Transcendence	**0.797**	0.022	*0.364*

Identity	**0.660**	0.141	*0.544*

Value 1	0.250	**0.786**	*0.319*

Value 2	-0.027	**0.877**	*0.231*

The Kaiser-Meyer-Olkin measure of sampling adequacy (0.65) confirmed that partial correlations are high enough to perform a factor analysis. Moreover, uniqueness values (giving the proportion of the common variance of the variable not associated with the factors) for each variable loaded on the retained factors (all < 0.6) were low enough to validate the use of factorial analysis in this context.

### Reliability assessment

Inter-item correlations and item-to-total correlations are reported in Table 5. The scale's internal consistency was acceptable (Cronbach α = 0.60), considering the number of items in the test, and the fact that our construct is not unidimensional [29].

**Table 5:** Inter-item and item-to-total correlations

	Meaning	Transcendence	Identity	Value 1	Value 2	SDAT total
**Meaning**	1					

**Transcendence**	0.28^†^	1				

**Identity**	0.20*	0.27^†^	1			

**Value 1**	0.26^†^	0.21*	0.24^†^	1		

**Value 2**	0.19*	0.11	0.13	0.41^†^	1	

**SDAT Total**	**0.57**^†^	**0.61**^†^	**0.61**^†^	**0.66**^†^	**0.59**^†^	**1**

*: P-value < 0.01

^†^: P-value < 0.001

No mention: P-value > 0.05

Intra-rater reliability at 3 months was high for the three judges. Intraclass correlation coefficients of the 3 judges were 0.95 (95% CI: 0.85-1.0), 0.96 (95% CI: 0.87-1.0) and 0.96 (95% CI: 0.87-1.0), respectively. Intra-rater agreement about the presence versus absence of spiritual distress was perfect (100%).

Inter-rater reliability coefficient (N = 21 SDAT interviews and two judges) was high (0.87), and Cohen's kappa for spiritual distress was 90.4 (agreement: 95.4%).

### Validity assessment

Assessment of *criterion-related validity *showed that SDAT scores correlated significantly with the FACIT-Sp scores (Spearman's Rho = -0.45, P < .001) and with the scoring of the single question "Are you at peace?" (Spearman's Rho = -0.33, P < .001). This indicates that higher spiritual distress was associated with lower spiritual well-being, and less peacefulness, respectively.

*Concurrent validity *showed a significant positive correlation between the SDAT and GDS scores (Spearman Rho = 0.43, P < .001), indicating that higher spiritual distress was associated with more depressive symptoms. Moreover, compared with patients showing no severely unmet spiritual need, patients with at least one severe unmet spiritual need had higher odds of occurrence of a family meeting to determine discharge disposition (OR 5.3, 95%CI 2.0-13.8, P = .001). This relationship remained significant in multivariate analysis that adjusted for age, gender, functional and depressive status (adjOR 4.7, 95%CI 1.4-16.3, P = .02).

Analysis of *predictive validity *showed that SDAT total score was moderately correlated with patient's LOS (Spearman's Rho = 0.15, P = .03). However, this relationship did not remain significant in multivariable analysis. Finally, patients presenting at least one severely unmet spiritual need (45/203) were more often discharged to a nursing home (6/45) than were those without severely unmet spiritual need (6/158) (P = .027). The number of patients discharged to nursing home was too small to perform a multivariate analysis.

## Discussion

This study shows that the SDAT has acceptable to good internal consistency, as well as intra-rater and inter-rater reliability. Criterion-related and concurrent validity were also in the range considered as substantial [30]. Finally, the presence of at least one severely unmet need significantly predicted the occurrence of a family meeting to define discharge disposition, even when controlling for depressive symptoms. Overall, the psychometrics properties of the SDAT instrument appear good enough to support further investigation of its predictive validity.

These results deserve several comments. First, the assessment of the SDAT's internal consistency (Cronbach's α and inter-item correlations) showed only moderate correlations. This is not surprising given the concept used to develop this instrument. The spirituality construct underlying its development was explicitly multidimensional, including four distinct dimensions. High correlations between items measuring needs related to these four separate dimensions were therefore not expected. From a more technical stand point, high reliability coefficients would also have been surprising as they depend not only on item homogeneity, but also on their number in the scale, a number limited to five in the SDAT. Nevertheless, item-to-total correlations were highly significant, indicating that each item contributes additional specific information. This last appreciation is further supported by results of the factor analysis that clearly identified two main factors. The first factor (loading on Meaning, Transcendence and Identity spiritual needs) could be interpreted as reflecting the patient's intrinsic inner spirituality, while the second factor (loading on Values needs) would reflect the combined balance between the patient's and the health professional's system of values. Overall, these results show that the SDAT has acceptable psychometrics properties that make it a valid and reliable instrument to assess spiritual distress in older patients hospitalized in post-acute rehabilitation.

The second comment is related to the cut-off used to define spiritual distress. This cut-off was determined according to a clinical definition of spiritual distress. This cut-off is debatable and will probably have to be refined according to further sensitivity analyses of the instrument's predictive validity.

The final comment relates to the specific contribution of the present study to the field of spirituality research. Results from this study provide a preliminary estimate of the prevalence of spiritual distress in older patients hospitalized in post-acute rehabilitation. Overall, these results indirectly raise the question whether spiritual distress could be a neglected problem in these patients. Future studies need to investigate in more details the potential influence of spiritual distress on patients' health outcomes and quality of life. This is a necessary step to determine whether specific interventions targeting spiritual distress should be developed and tested in the future.

This study has some limitations. Test-retest assessment would have been more accurate if the SDAT interviews could have been repeated. However, this option was considered inappropriate because two successive interviews investigating intimate concerns were considered too demanding in this vulnerable population. In addition, the current lack of knowledge on the dynamic of spiritual state when undergoing post-acute rehabilitation would have made it difficult to choose a time window both large enough to avoid recall bias in the interviewer and tight enough to minimize the potential effect of numerous factors that could influence these patients' spiritual state. Alternatively, using a different interviewer would have resulted in investigating inter-rater agreement at the same time, a clearly unsatisfying option. Video-taped interviews and measures of intra-rater reliability were therefore preferred. Nevertheless, only four interviews were videotaped and analyzed by three judges and this limited number of cases limits also the statistical power analysis.

Refusal rate (31.8%) is another limitation of this study. Patients showed some reluctance to participate in the study as most considered they had already answered too many questions on admission. However, comparison of the characteristics of participants and refusers did not suggest differences that could have affected results. Finally, additional limitations concerning the sampling bias exist. Subjects suffering from significant cognitive impairment or considered too ill to complete the interview were excluded from the study. This sampling bias might have influenced the results and underestimated the prevalence of spiritual distress in this population of older hospitalized patients.

This study also has clear strengths. The SDAT underwent an extensive validation process. Most instruments currently available to assess spirituality have not undergone such a rigorous and complete validation process [19]. Previous work showed good face validity and acceptability [23]. This study further extends documentation of the SDAT properties by showing its reliability and validity to assess spiritual distress in older hospitalized patients.

Another original contribution of this work is to propose a validated instrument based on a semi-structured interview rather than on a set of closed questions. The SDAT is unique in this regard as it offers the possibility to assess spirituality through an approach that is centered on the patient. Rigorous validation of such semi-structured interviews, as reported here, is uncommon.

## Conclusions

The SDAT instrument performed adequately to assess spiritual distress in older patients admitted to post-acute rehabilitation. Results also suggest that spiritual distress is frequent in these patients and suggest the need for further investigation to identify those most at risk. These are preliminary steps to determine more precisely the potential benefits to be obtained from interventions aiming at spiritual support in older patients experiencing distress.

## Competing interests

The authors declare that they have no competing interests.

## Authors' contributions

SM planned the study, supervised the validation of the instrument and wrote the paper. EM supervised the choice of psychometric analysis and performed all statistical analyses. BS contributed to the psychometric properties analysis and revised the manuscript. ER helped to performed validation of the SDAT. CB conceptualized the overall methodology and revised the manuscript. All authors read and approved the final manuscript.

## Pre-publication history

The pre-publication history for this paper can be accessed here:

http://www.biomedcentral.com/1471-2318/12/13/prepub

## References

[B1] Mueller PS Plevak DJ Rummans TA Religious involvement, spirituality, and medicine: implications for clinical practice Mayo Clin Proc 2001 76 1225 1235 10.4065/76.12.1225 11761504

[B2] Balboni TA Paulk ME Balboni MJ Phelps AC Loggers ET Wright AA Block SD Lewis EF Peteet JR Prigerson HG Provision of spiritual care to patients with advanced cancer: associations with medical care and quality of life near death J Clin Oncol 2010 28 445 452 10.1200/JCO.2009.24.8005 20008625 PMC2815706

[B3] Krause N Religious meaning and subjective well-being in late life J Gerontol B Psychol Sci Soc Sci 2003 58 3 S160 S170 10.1093/geronb/58.3.S160 12730317

[B4] Idler EL Kasl SV Religion among disabled and nondisabled persons II: attendance at religious services as a predictor of the course of disability J Gerontol Soc Sci 1997 52 6 S306 S316 10.1093/geronb/52b.6.s306 9403524

[B5] Wink P Scott J Does religiousness buffer against the fear of death and dying in late adulthood? Findings from a longitudinal study J Gerontol B Psychol Sci Soc Sci 2005 60 207 214 10.1093/geronb/60.4.P207 15980288

[B6] Crowther MR Parker MW Achenbaum WA Larimore WL Koenig HG Rowe and Kahn's model of successful aging revisited: positive spirituality - the forgotten factor Gerontologist 2002 42 613 620 10.1093/geront/42.5.613 12351796

[B7] Chally PS Carlson JM Spirituality, rehabilitation, and aging: a literature review Arch Phys Med Rehabil 2004 85 S60 S65 15221731 10.1016/j.apmr.2004.03.013

[B8] Silvestri GA Knittig S Zoller JS Nietert PJ Importance of faith on medical decisions regarding cancer care J Clin Oncol 2003 21 7 1379 1382 10.1200/JCO.2003.08.036 12663730

[B9] Lo B Ruston D Kates LW Arnold RM Cohen CB Faber-Langendoen K Pantilat SZ Puchalski CM Quill TR Rabow MW Schreiber S Sulmasy DP Tulsky JA Discussing religious and spiritual issues at the end of life: a practical guide for physicians JAMA 2002 287 749 754 10.1001/jama.287.6.749 11851542

[B10] Koenig HG McCullough ME Larson DB Handbook of religion and health 2001 New York: Oxford University Press

[B11] Koenig HG George LK Titus P Religion, spirituality, and health in medically ill hospitalized older patients J Am Geriatr Soc 2004 52 554 562 10.1111/j.1532-5415.2004.52161.x 15066070

[B12] Pargament KI Koenig HG Tarakeshwar N Hahn J Religious coping methods as predictors of psychological, physical and spiritual outcomes among medically ill elderly patients: a two-year longitudinal study J Health Psychol 2004 9 713 730 10.1177/1359105304045366 15367751

[B13] McClain CS Rosenfeld B Breitbart W Effect of spiritual well-being on end-of-life despair in terminally-ill cancer patients Lancet 2003 361 1603 1607 10.1016/S0140-6736(03)13310-7 12747880

[B14] Pargament KI Koenig HG Tarakeshwar N Hahn J Religious struggle as a predictor of mortality among medically ill elderly patients: a 2-year longitudinal study Arch Intern Med 2001 161 15 1881 1885 10.1001/archinte.161.15.1881 11493130

[B15] Astrow AB Wexler A Texeira K He MK Sulmasy DP Is failure to meet spiritual needs associated with cancer patients' perceptions of quality of care and their satisfaction with care? J Clin Oncol 2007 25 5753 5757 10.1200/JCO.2007.12.4362 18089871

[B16] Puchalski C Ferrell B Virani R Otis-Green S Baird P Bull J Chochinov H Handzo G Nelson-Becker H Prince-Paul M Pugliese K Sulmasy D Improving the Quality of Spiritual Care as a Dimension of Palliative Care: The Report of the Consensus Conference J Palliat Med 2009 12 885 904 10.1089/jpm.2009.0142 19807235

[B17] Sulmasy DP A biopsychosocial-spiritual model for the care of patients at the end of life Gerontologist 2002 42 Special Issue 1 24 33 12415130 10.1093/geront/42.suppl_3.24

[B18] Brennan M Heiser D Introduction: Spiritual Assessment and Intervention: Current Directions and Applications J Religion Spirituality Aging 2004 17 1 20 10.1300/J496v17n01_01

[B19] Monod S Brennan M Rochat E Martin E Rochat S Bula CJ Instruments Measuring Spirituality in Clinical Research: A Systematic Review J Gen Intern Med 2011 doi:10.1007/s11606-011-1769 10.1007/s11606-011-1769-7 PMC3208480 21725695

[B20] Peterman AH Fitchett G Brady MJ Hernandez L Cella D Measuring spiritual well-being in people with cancer: The Functional Assessment of Chronic Illness Therapy-Spiritual Well-Being Scale (FACIT-Sp) Ann Behav Med 2002 24 49 58 10.1207/S15324796ABM2401_06 12008794

[B21] Daaleman TP Frey BB The Spirituality Index of Well-Being: A New Instrument for Health-Related Quality-of-Life Research Ann Fam Med 2004 2 499 503 10.1370/afm.89 15506588 PMC1466734

[B22] Monod S Rochat E Bula C Spencer B The Spiritual Needs Model: Spirituality Assessment in the Geriatric Hospital Setting J Religion Spirituality Aging 2010 22 271 282 10.1080/15528030.2010.509987

[B23] Monod SM Rochat E Bula CJ Jobin G Martin E Spencer B The Spiritual Distress Assessment Tool: An instrument to assess spiritual distress in hospitalised elderly persons BMC Geriatr 2010 10 88 10.1186/1471-2318-10-88 21144024 PMC3017043

[B24] Steinhauser KE Voils CI Clipp EC Bosworth HB Christakis NA Tulsky JA "Are you at peace?": one item to probe spiritual concerns at the end of life Arch Intern Med 2006 166 1 101 105 10.1001/archinte.166.1.101 16401817

[B25] Yesavage JA Brink TL Rose TL Lum O Huang V Adey M Leirer VO Development and validation of a geriatric depression screening scale: a preliminary report J Psychiatr Res 1982 17 37 49 10.1016/0022-3956(82)90033-4 7183759

[B26] Folstein MF Folstein SE McHugh PR "Mini-mental state". A practical method for grading the cognitive state of patients for the clinician J Psychiatr Res 1975 12 189 198 10.1016/0022-3956(75)90026-6 1202204

[B27] Monod S Rochat E Martin E Bula C Spiritual assessment in older patients undergoing post-acute rehabilitation: A pilot study Gerontologist 2007 47 Special Issue I 774

[B28] Katz S Assessing self-maintenance: activities of daily living, mobility, and instrumental activities of daily living J Am Geriatr Soc 1983 31 721 727 6418786 10.1111/j.1532-5415.1983.tb03391.x

[B29] Schmitt N Uses and abuses of coefficient alpha Psychol Assess 1996 8 350 353

[B30] Stewart AL Wonca Classification Committee Psychometric Considerations in Functional Status Instruments Functional Status Measurement in Primary Care 1990 New York: Springer 3 26

